# Seroprevalence and Molecular Analysis of Bovine Leukemia Virus in Kazakhstan

**DOI:** 10.3390/v17070956

**Published:** 2025-07-07

**Authors:** Saltanat Mamanova, Ainur Nurpeisova, Elvira Bashenova, Saira Kaimoldina, Vladimir Kirpichenko, Perizat Akshalova, Aiken Karabassova, Malik Yussupov, Akzhigit Mashzhan, Dauriya Tazhbayeva, Zhandos Abay, Marzena Rola-Luszczak, Jacek Kuzmak, Raikhan Nissanova, Markhabat Kassenov

**Affiliations:** 1 Kazakh Scientific Research Veterinary Institute, LLP, 223 Rayymbek Avenue, Almaty 050016, Kazakhstan; mamanovasaltanat248@gmail.com (S.M.); nurai1005@gmail.com (A.N.); elvirabashenova17@gmail.com (E.B.); kaimoldina84mir@gmail.com (S.K.); vladimir.kirpichenko1992@gmail.com (V.K.); peri.akshalova@gmail.com (P.A.); aiken.karabasova@gmail.com (A.K.); yussupovmalik@gmail.com (M.Y.); abai.zhandos15@gmail.com (Z.A.); kasenovmarhabat@gmail.com (M.K.); 2 Almaty Branch of the National Center for Biotechnology, 14 Zhahanger St., Almaty 050054, Kazakhstan; aj.akzhigit@gmail.com; 3 Institute of Veterinary and Agrotechnology, Zhangir Khan West Kazakhstan Agrarian Technical University, 51 Zhangir Khan Street, Uralsk 090009, Kazakhstan; dauriyatazhbaeva@gmail.com; 4 WOAH Reference Laboratory for Enzootic Bovine Leukosis, National Veterinary Research Institute, Partyzantów 57 Avenue, 24-100 Puławy, Poland; mrolka@piwet.pulawy.pl (M.R.-L.); jkuzmak@piwet.pulawy.pl (J.K.)

**Keywords:** bovine leukemia virus, Kazakhstan, epizootic dynamics, molecular epidemiology

## Abstract

Bovine leukemia virus (BLV) remains a major concern for cattle industries worldwide due to its persistent nature, economic impact, and challenges in control. In this study, we conducted a comprehensive nationwide survey of BLV in Kazakhstan between 2014 and 2024, utilizing serological diagnostics to assess prevalence and characterize viral genotypes (2024). A total of 433,537 serum samples were screened by agar gel immunodiffusion (AGID), revealing an overall seroprevalence of 5.87%, with the highest rates observed in the North Kazakhstan, Kostanay, and East Kazakhstan regions. In 2024, a targeted analysis of 3736 serum and 536 whole blood samples across 17 regions was performed using AGID, ELISA, real-time PCR, and nested PCR. ELISA demonstrated higher sensitivity than AGID (10.4% vs. 8.2%), confirmed by statistical correlation (r = 0.97, *p* < 0.001) and a Wilcoxon signed-rank test (*p* = 0.026). Real-time PCR detected BLV DNA in 4.7% of samples, with the highest positivity in the East Kazakhstan and Abai regions, confirming active viral circulation. Validation of a domestically developed AGID diagnostic kit showed full concordance with commercial assays (IDEXX, IDvet), supporting its use in national surveillance programs. These findings highlight the endemic status of BLV in Kazakhstan. Molecular analysis of sequenced isolates revealed the presence of genotype G-7, consistent with strains circulating in neighboring countries. Together, these results underscore the importance of integrated serological and molecular approaches for effective monitoring and control.

## 1. Introduction

Bovine leukemia virus (BLV), a member of the genus *Deltaretrovirus* (family *Retroviridae*), is the causative agent of enzootic bovine leukosis (EBL)—a chronic infectious disease with global economic impact due to reduced productivity, reproductive losses, early culling, and trade restrictions [1]. Despite global control programs, BLV remains endemic in several regions, including parts of Asia, North America, and Eastern Europe [1,2].

Kazakhstan, with its large cattle population and agricultural economy, is particularly vulnerable to the impacts of BLV. However, the country’s epidemiological data on BLV are limited, impeding a full understanding of the virus’s distribution and consequences for animal health and productivity [3].

Serological surveillance is essential for identifying high-risk herds and informing control strategies. The agar gel immunodiffusion (AGID) assay, recommended by the World Organisation for Animal Health (WOAH), is widely used for BLV antibody detection due to its specificity, affordability, and suitability for large-scale screening in resource-limited regions [4].

BLV prevalence is variable globally. In developed countries, it ranges from 30% to 80% in dairy herds, while developing countries often report higher prevalence due to inadequate control resources [5,6,7].

The economic impact of BLV is multifaceted. Infected cattle, particularly those in the subclinical stage, may exhibit reduced milk yield, which contributes to economic losses in dairy production systems. Recent studies have shown that the severity of BLV-associated production losses is modulated by the animal’s genetic background, especially polymorphisms in the bovine leukocyte antigen (BoLA)-DRB3 gene. Cattle carrying susceptible alleles (e.g., BoLA-DRB3*015:01 and 012:01) tend to develop higher proviral loads (PVLs), which are associated not only with increased viral transmission but also with greater impacts on milk yield and immune function. Conversely, cattle with resistant alleles (e.g., BoLA-DRB3009:02 and *014:01:01) typically maintain lower PVL, experience milder immunopathological effects, and demonstrate better production performance. These findings underscore the importance of incorporating host genetics into BLV control and selective breeding programs [8,9,10,11,12]. Furthermore, the progression to malignant lymphoma or leukemia in a small percentage of infected animals results in direct losses due to mortality and the costs associated with diagnosis and treatment.

BLV is primarily transmitted through direct contact with infected biological fluids, such as blood, milk, and colostrum [11]. Iatrogenic spread via contaminated instruments and vertical transmission from dam to calf further complicate control efforts [12]. Several risk factors have been identified as contributing to the spread of BLV, including herd size, management practices, and the presence of persistently infected animals [13].

Despite the importance of serological surveillance, comprehensive seroprevalence data for BLV in Kazakhstan remain scarce. Existing studies have documented only sporadic cases without providing a clear picture of the overall seroprevalence and geographical distribution. Such limited data hinder efforts to quantify the true economic and health burden of BLV in the Kazakhstani cattle industry.

Large dairy herds with intensive management systems are particularly vulnerable to BLV transmission due to the frequent handling of animals and the use of shared equipment. Additionally, the lack of effective vaccines and the high cost of diagnostic testing have hindered efforts to control the disease in many regions.

The virus exhibits considerable variability in its genome, particularly in the *env* gene, which encodes the gp51 envelope glycoprotein [14,15]. This genetic variability has implications for diagnostic accuracy, vaccine development, and the design of control strategies. Recent studies have identified multiple BLV genotypes circulating globally, with genotype 1 being the most prevalent. However, the genetic diversity of BLV in Kazakhstan remains characterized only in some regions of the country, highlighting the need for comprehensive molecular characterization.

Over the past decade, advancements in diagnostic technologies have significantly improved the detection and characterization of BLV [16]. Molecular techniques, including conventional PCR, real-time PCR (qPCR), and next-generation sequencing (NGS), have enabled researchers to quantify viral load, identify genetic variants, and monitor the spread of the virus. Despite technological advances, large-scale BLV surveillance remains difficult in resource-limited settings due to high diagnostic costs, specialized equipment needs, and a shortage of trained personnel [17,18].

Kazakhstan’s livestock sector is a cornerstone of its agricultural economy, with cattle farming playing a central role in rural livelihoods and food security [19]. However, the lack of comprehensive data on BLV prevalence and distribution in the country has hindered efforts to assess the virus’s impact on cattle health and productivity. Previous studies have reported some cases of BLV in Kazakhstan, but these have been limited in scope and do not provide a clear picture of the virus’s epizootic dynamics [20]. Kazakhstan’s diverse geography, climate, and cattle management practices create a complex setting for BLV transmission. Identifying context-specific drivers of spread is crucial for designing effective control strategies tailored to local challenges.

This study aims to evaluate BLV seroprevalence across Kazakhstan using the AGID method, perform molecular characterization of circulating strains, and validate a domestically produced AGID test. These results will support improved disease monitoring and inform national control strategies.

## 2. Materials and Methods

### 2.1. Study Population Characteristics

This study was conducted across 17 regions of Kazakhstan, covering both dairy and beef cattle farms. A total of 433,537 serum samples and 536 whole blood samples were collected from cattle aged 1 to 10 years. The sampling strategy was designed to ensure representation of different production systems (intensive, semi-intensive, and extensive) and herd sizes (ranging from smallholder farms to large commercial operations).

### 2.2. Study Design

This retrospective study analyzed the epizootic dynamics of BLV in Kazakhstan over a 11-year period (2014–2024). The dataset covered annual surveillance from 2014 to 2024. Although the data were collected over time, this study focused on cumulative seroprevalence at the national and regional levels. A detailed temporal analysis is planned for future work. This study integrated serological testing by AGID.

In 2024, molecular testing (real-time PCR, nested PCR) and sequencing were performed to characterize current viral circulation and genotypes.

### 2.3. Sampling

The minimum sample size for the serological surveillance of bovine leukemia was calculated using the formula presented by Thrusfield [21]. Blood samples were collected from the jugular vein using sterile vacuum tubes (Vacutainer^®^, BD). For serological analysis, blood was collected in tubes without anticoagulant, allowed to clot at room temperature for 30–60 min, and then centrifuged at 3000× *g* for 10 min to separate the serum. Serum aliquots were stored at −20 °C until analysis. For molecular studies, blood was collected in EDTA-coated tubes, stored at +4 °C, transported to the laboratory within 24–48 h. Leukocyte fractions were used for antigen preparation, while whole blood samples were used for genomic DNA extraction. Each sample was assigned a unique identification number, including the animal ID, farm location, and date of collection.

### 2.4. Laboratory Methods

Agar gel immunodiffusion (AGID) tests were performed using commercial kits containing BLV gp51 antigen (IDEXX Laboratories, Montpellier, France), as well as a locally developed AGID kit from KazSRVI (Almaty, Kazakhstan), in accordance with the manufacturers’ protocols. The BLV antigen used in the locally developed AGID kit was prepared from persistently infected FLK-BLV cell cultures. The cell suspension was placed in semipermeable dialysis bags and concentrated by passive osmotic extraction using external application of dry polyethylene glycol 6000 (PEG-6000) at 4 °C for 24 h. The concentrated antigen was clarified by low-speed centrifugation and stored at −20 °C. The functionality of the antigen was confirmed by its ability to produce clear and specific precipitation lines in AGID when tested against reference positive sera obtained during the WOAH Twinning Project in accordance with the WOAH Manual of Diagnostic Tests and Vaccines for Terrestrial Animals [22]. Validation of the KazSRVI AGID kit was carried out in parallel with two commercial kits (IDEXX Laboratories, Montpellier, France; IDvet, Grabels, France) using a panel of reference serum samples obtained during the Twinning project from the WOAH reference laboratory (NVRI, Pulawy, Poland).

BLV-specific antibodies were detected using the IDEXX Leukosis Blocking Ab Test (IDEXX Laboratories, France; Catalog No. 06-02140-08), a blocking ELISA designed to detect antibodies against the gp51 protein of BLV in bovine serum or plasma. Optical density (OD) values were measured at 450 nm using a microplate reader (Multiskan FC, Thermo Fisher Scientific, Vantaa, Finland). Samples with OD values above the cutoff value established by the manufacturer (S/P ≥ 0.5) were classified as positive.

Genomic DNA was extracted from whole blood using the QIAamp DNA Blood Mini Kit (QIAGEN GmbH, Hilden, Germany) following the manufacturer’s instructions. DNA concentration and purity were assessed using a NanoDrop spectrophotometer (Thermo Fisher Scientific, Waltham, MA, USA).

Nested PCR targeting the BLV *env* gene (encoding gp51) was performed in two rounds. In the first round, amplification was carried out using the primers env5032 (5′-TCTGTGCCAAGTCTCCCAGATA-3′) and env5608 (5′-AACAACAACCTCTGGGAAGGGT-3′), generating a 600 bp product. Reactions were performed in a total volume of 50 µL, containing 8 µL of 5× ScreenMix (Evrogen, Moscow, Russia), 1.5 µL of each primer (20 µM), 5 µL of template DNA, and nuclease-free water. The cycling conditions were as follows: initial denaturation at 94 °C for 2 min; 39 cycles of 95 °C for 30 s, 62 °C for 30 s, and 72 °C for 60 s; followed by a final extension at 72 °C for 4 min. For the second round, nested PCR was conducted using the primers env5099 (5′-CCCACAAGGGGGCGCCGGTTT-3′) and env5521 (5′-GCGAGGCCGGGTCCAGAGCTGG-3′), yielding a 444 bp product. The nested PCR method and primer sets were originally described and validated by Fechner et al. [23]. The reaction mixture (50 µL total) contained 7 µL of 5× ScreenMix, 1.5 µL of each primer (20 µM), 7 µL of the first-round PCR product, and nuclease-free water. Cycling conditions were as follows: initial denaturation at 94 °C for 2 min; 39 cycles of 95 °C for 30 s, 70 °C for 30 s, and 72 °C for 60 s; with a final extension at 72 °C for 4 min. Amplification products were analyzed by electrophoresis on 1.5% agarose gels stained with ethidium bromide and visualized under UV illumination.

Real-time PCR detection of BLV was performed using the QuantiTect Multiplex PCR No ROX Kit (QIAGEN GmbH, Hilden, Germany). The assay targeted a conserved region within pol gene of the BLV genome, using primers MRF (5′-CCTCAATTCCCTTTAAACTA-3′), MRR (5′-GTACCGGGAAGACTGGATTA-3′), and probe MRBLV (5′-6FAM-GAACGCCTCCAGGCCCTTCA-BHQ1-3′), as described by Rola-Łuszczak et al. [24]. This method has been validated and widely used for the sensitive detection of BLV proviral DNA in infected cattle. Amplification was carried out on a Rotor-Gene Q thermocycler (QIAGEN GmbH, Hilden, Germany) with the following cycling conditions: initial activation at 96 °C for 10 min, followed by 37 cycles of denaturation at 94 °C for 45 s, annealing at 58 °C for 1 min, and extension at 72 °C for 1 min, with a final extension at 72 °C for 7 min. Fluorescence signals were collected during the annealing step in each cycle.Sequencing of nested —PCR products were purified using the GeneJET PCR Purification Kit (Thermo Fisher Scientific, Waltham, MA, USA) and sequenced bidirectionally at the National Center for Biotechnology (Almaty, Kazakhstan) using a GA3500 Genetic Analyzer (Applied Biosystems, Foster City, CA, USA). Sanger sequencing of BLV DNA fragments was performed using the BigDye Terminator v3.1 Cycle Sequencing Kit (Thermo Fisher Scientific, Waltham, MA, USA) following the manufacturer’s protocol. Reaction tubes containing the sequencing mixture were placed in a preheated thermal cycler at 96 °C, and the following cycling conditions were applied: denaturation at 96 °C for 20 s, primer annealing at 50 °C for 10 s, and extension at 60 °C for 4 min. Post-sequencing purification was carried out using the BigDye XTerminator Purification Kit (Thermo Fisher Scientific, Waltham, MA, USA). Sequence alignment was performed using ClustalW in MEGA version 11.0 [25]. Phylogenetic trees were constructed using the maximum likelihood method under the Tamura–Nei model with 1000 bootstrap replicates. Reference sequences from GenBank were used for genotype identification [26,27,28].

### 2.5. Statistical Analysis

Data were analyzed using SPSS v.26 (IBM, Armonk, NY, USA) and R v.4.2.1. Seroprevalence was calculated as the proportion of positive samples with 95% confidence intervals (CIs). The sensitivity and specificity of ELISA were determined relative to the AGID as the reference method. Cohen’s kappa coefficient (κ) was used to assess agreement between serological and molecular methods.

### 2.6. Ethical Considerations

The study protocol was approved by the Bioethics Committee of the KazSRVI, Ministry of Health of the Republic of Kazakhstan (Protocol No. 5-2020). All procedures were conducted in accordance with the World Organisation for Animal Health (WOAH) guidelines for animal welfare [29]. Blood sampling was performed by trained veterinarians to minimize stress and discomfort to the animals.

### 2.7. Quality Control

To ensure the accuracy and reliability of laboratory results, stringent quality control measures were implemented throughout the study. Equipment Calibration Laboratory instruments, including centrifuges, PCR machines, and microplate readers, were calibrated monthly according to manufacturer guidelines.

### 2.8. Control Samples

Each test lot included positive and negative controls. Positive controls consisted of known BLV-positive serum Q19 (calibrated against WOAH standard, EO5) or DNA (samples provided by the WOAH reference laboratory for EBL, NVRI, Puławy, Poland), while negative controls included samples from BLV-negative animals and a no-template control (NTC).

## 3. Results

To provide a comprehensive overview of the epidemiological situation of BLV in Kazakhstan, we first present long-term serological monitoring data collected from 2014 to 2024 across multiple regions. This is followed by an evaluation of the performance of the local AGID assay using a subset of recent samples, which confirms the utility of the in-house method for serological diagnosis.

### 3.1. Seroprevalence of BLV in Cattle in Kazakhstan (2014–2024)

A comprehensive serological survey of BLV in cattle was conducted in Kazakhstan between 2014 and 2024. As summarized in Table 1, a total of 433,537 serum samples were collected and tested by commercial AGID, of which 25,450 (5.87%) yielded positive results.

Over the 10-year monitoring period, the highest BLV seroprevalence was recorded in North Kazakhstan (16.79%), Kostanay (13.56%), and East Kazakhstan (8.39%), indicating persistent endemicity. Conversely, minimal or negligible positivity was observed in the Kyzylorda, Mangystau, and Zhetysu regions, suggesting either effective regional control strategies or limited virus circulation. National prevalence showed sharp fluctuations over the study period, with peaks observed in different regions. These findings highlight pronounced regional disparities and underscore the need for continued serological monitoring and targeted preventive measures.

### 3.2. Serological Mapping and Test Performance Comparison for BLV (2024)

**Figure 1:**
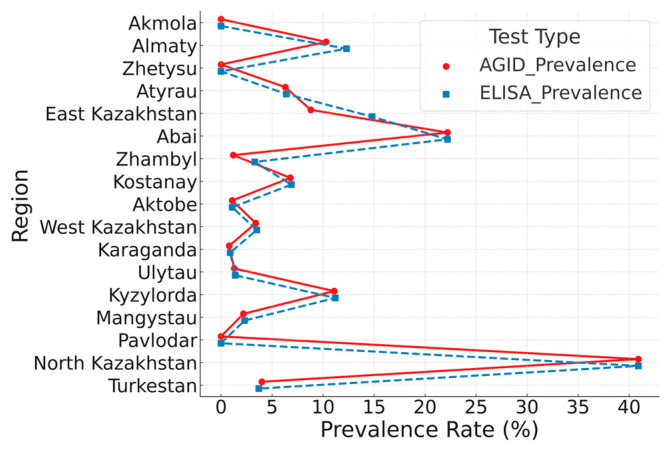
Paired comparison of bovine leukemia virus (BLV) seroprevalence rates in 17 regions of Kazakhstan in 2024, as determined by AGID and ELISA.

In 2024, researchers at the Kazakh Research Institute of Veterinary Science conducted serological (AGID, ELISA) and molecular (real-time PCR, nested PCR) analyses on 3736 serum samples and 536 whole blood samples collected from various herds across 17 regions of the Republic. The results of AGID and ELISA testing for bovine leukemia are presented in Figure 1.

Each line connects prevalence rates obtained by both methods for a given region. A strong positive correlation was observed (Pearson’s r = 0.97, *p* < 0.001). Statistical comparison using the Wilcoxon signed-rank test revealed a significant difference between methods (*p* = 0.026), with ELISA consistently showing higher prevalence rates. While this may suggest increased sensitivity, it could also reflect reduced specificity. Therefore, conclusions regarding diagnostic performance should be drawn cautiously. To explore this, a subset of ELISA-positive/AGID-negative samples was analyzed by real-time PCR. Detection of BLV proviral DNA in several of these samples supports the interpretation that ELISA may identify additional true positive cases undetected by AGID. These findings underscore the superior sensitivity of ELISA and its value as a complementary tool alongside AGID in large-scale BLV surveillance.

**Figure 2:**
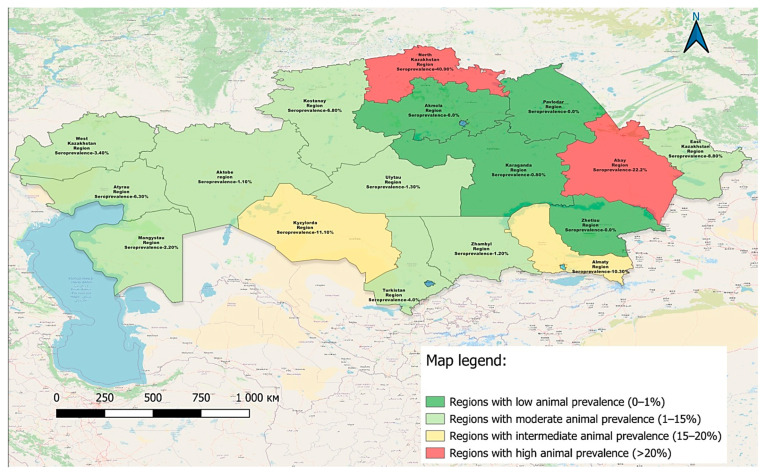
Geographic distribution of BLV-positive cattle in Kazakhstan in 2024 based on AGID testing.

The spatial distribution of BLV seropositive cases identified by AGID testing in 2024 is shown in Figure 2, highlighting regional variation in seroprevalence and indicating potential areas of increased infection tendency.

The map illustrates the geographical distribution of BLV seropositive cases identified during the 2024 surveillance campaign. In total, more than 3700 serum samples were tested across 17 regions of the Republic of Kazakhstan using an AGID assay, which is recommended by the WOAH as the primary screening method.

The results of this serological monitoring are summarized in Table 2, providing an overview of infection prevalence by region and highlighting areas with elevated epizootic risk.

These regional findings, based on AGID screening, reflect pronounced differences in BLV seroprevalence across Kazakhstan. Notably, the North Kazakhstan, Abai, and Kyzylorda regions demonstrated the highest positivity rates, indicating persistent endemicity and elevated epizootic pressure. In contrast, several regions reported no positive samples, which may reflect either limited virus circulation or under detection. These results underscore the need for both reliable diagnostic tools and region-specific surveillance strategies.

To address this need, a domestically developed AGID diagnostic kit was validated as part of the current study to support nationwide BLV monitoring and control efforts.

### 3.3. Diagnostic Validation of the KazSRVI AGID Test

Comparative testing using a total of 701 serum samples collected from cattle of various breeds and age groups was conducted to evaluate the diagnostic performance of the KazSRVI AGID kit. The results were compared with two commercially available AGID systems (IDEXX and IDvet). Full diagnostic concordance was observed across all test platforms. As part of the present study, a domestic AGID-based diagnostic reagent for the detection of BLV-specific antibodies was developed by researchers at the Kazakh Scientific Research Veterinary Institute (KazSRVI). The test system was intended for use in routine serological surveillance and laboratory confirmation of BLV infection in field conditions. Validation involved side-by-side comparison with commercial test kits using well-characterized serum panels. The results of this comparative evaluation are presented in Table 3. The observed 100% agreement between the KazSRVI kit and the commercial systems supports its reliability and suitability for inclusion in national surveillance programs.

The results presented in Table 3 demonstrate complete agreement between the KazSRVI AGID kit and the two commercial AGID kits (ID VET and IDEXX) across all sampling locations. The high level of concordance observed in this study provides strong evidence for the reliability of the KazSRVI AGID test as a practical alternative to imported commercial kits for the serological detection of BLV antibodies. Given its local production, cost-effectiveness, and diagnostic performance, the KazSRVI AGID test represents a valuable tool for large-scale surveillance and control programs aimed at managing enzootic bovine leukosis in Kazakhstan.

### 3.4. Molecular Detection and Genetic Characterization of BLV Isolates in 2024

In addition to serological testing, molecular genetic analyses were performed on 536 whole blood samples collected from cattle across 17 regions of Kazakhstan. These samples were selected based on prior serological results obtained during the 2024 surveillance campaign. Specifically, the panel included (i) animals that tested positive by AGID and/or ELISA to confirm active infection; (ii) seronegative animals from high-prevalence farms to identify potential latent or early-stage infections; and (iii) a small number of randomly selected samples from regions with unknown or low seroprevalence to assess potential silent circulation. The results of real time PCR testing, along with the corresponding AGID and ELISA outcomes for the same 536 samples, are summarized in Table 4 to enable cross-method comparison.

The comparative analysis of real time PCR, AGID, and ELISA testing (Table 4) revealed that 25 out of 536 whole blood samples (4.7%) were positive for BLV proviral DNA. Serological testing of the same samples by AGID and ELISA yielded 89 (16.6%) and 93 (17.4%) positive results, respectively. Notably, the East Kazakhstan (15/27; 55.6%) and Abai (5/26; 19.2%) regions accounted for 80% of the PCR-confirmed cases, indicating active viral circulation and suggesting potential epidemiological hotspots. Several ELISA-positive but AGID-negative samples from these regions were also confirmed by PCR, supporting the added value of ELISA for detecting early or low-titer infections.

The highest number of BLV-positive samples was detected in East Kazakhstan Region, where three farms—Kamynshenskoe (Shemonaiha District), Bagration, and Ukrainka (Ulan District)—recorded five, six, and four positive cases, respectively. In Almaty Region, 3 positive cases (2.7%) were identified out of 110 animals tested at Mrnabayev Farm. In Abai Region, two farms—Lana 2 (Glukhovsky Rural District) and Mukinov (Ermazarsky Rural District)—reported 4 and 1 positive cases, respectively, out of 26 animals tested. In Karaganda Region, 2 positive cases (8.3%) were found among 24 samples collected from Bakhyt Farm in Bukhar-Zhyrau District. These results confirm the ongoing circulation of BLV in several regions of Kazakhstan and highlight the importance of molecular confirmation of serological findings.

**Figure 3:**
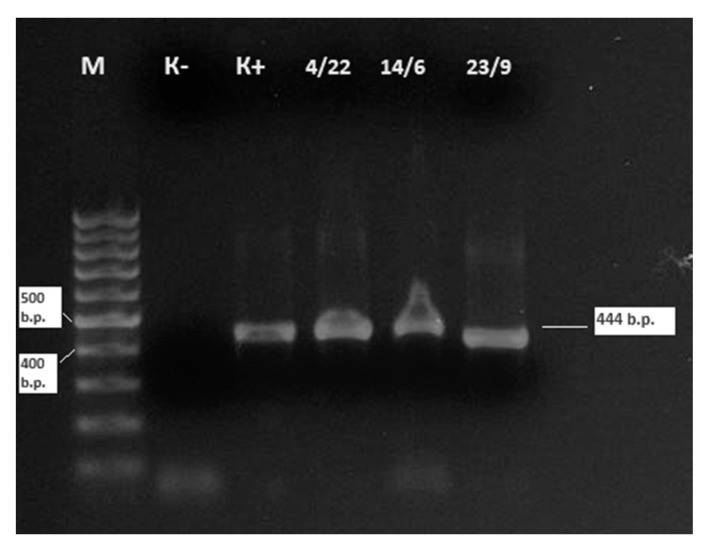
Electrophoresis results of nested PCR amplification targeting the *env* gene fragment of BLV from three field samples collected in East Kazakhstan Region. Note: M—molecular weight marker (1 kb DNA ladder); K−—negative control (no template control); K+—positive control (reference BLV DNA); 4/22(4), 14/6(14), 23/9(20)—field samples tested positive for BLV by nested PCR. Amplified products of ~444 bp confirm the presence of BLV *env* gene in all three samples. Samples 4 and 14 were collected from Kamyshenskoye Farm (Shemonaikha District, Vavilon rural area), and sample 20 was collected from Ukrainka Farm (Ulan District, Tokhtarov rural area). The intensity of the bands correlates with viral DNA load as inferred from real-time PCR Ct values (ranging from 27.1 to 29.0), indicating moderate-to-high proviral load in these animals.

**Figure 4:**
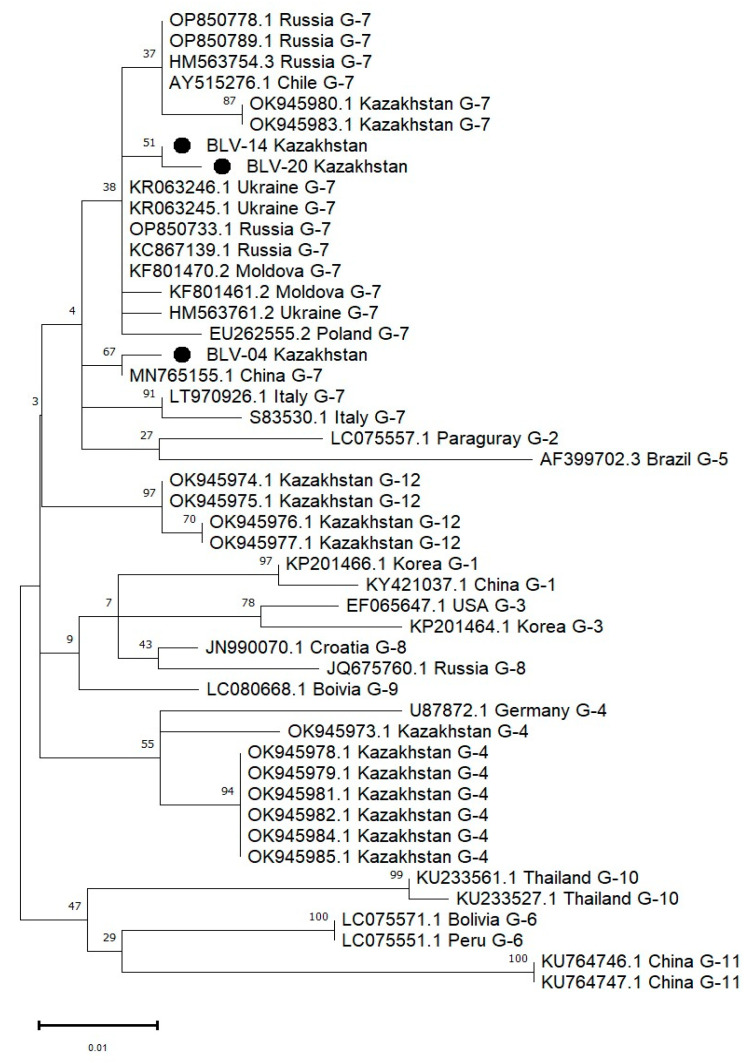
Phylogenetic tree of bovine leukemia virus (BLV) isolates circulating in Kazakhstan in 2024, with genotype classification ranging from G1 to G12. The tree was constructed using the maximum likelihood method under the Tamura–Nei model based on a 299 bp fragment of the env gene. The analysis included 42 nucleotide sequences. Codon positions included were 1st, 2nd, 3rd, and non-coding. All positions containing gaps and missing data were eliminated using the complete deletion option. Bootstrap values (>50%) based on 1000 replicates are shown next to the branches. Kazakhstani isolates are marked with solid circles (●). These sequences were compared with reference strains retrieved from GenBank, standardized by GenBank accession number, country of origin, and genotype.

For the sequencing of BLV isolates obtained in 2024, three positive samples with low cycle threshold (Ct) values in real-time PCR were selected. A classical nested PCR approach was used to amplify a specific fragment of the *env* gene, yielding a 444 bp product (Figure 3). These sequenced samples were all classified as belonging to genotype G-7 based on phylogenetic analysis (see Figure 4).

Samples No. 4 and 14 were obtained from whole blood of cattle at Kamyshenka Farm, Shemonaikha District, while sample No. 20 was obtained at Ukrainka Farm, Ulan District, all three samples from East Kazakhstan Region.

The phylogenetic relationship of the *env* gene fragment encoding the gp51 glycoprotein of BLV, identified in this study, was analyzed in comparison with 19 BLV sequences representing 12 different genotypes from various regions of the world.

The phylogenetic tree (Figure 4), constructed using the maximum likelihood (ML) method under the Tamura–Nei model, demonstrates that the BLV isolates obtained from cattle in Kazakhstan are distributed across three genotypes: G-4, G-7, and G-12. Specifically, isolates BLV-04, BLV-14, and BLV-20 cluster within genotype G-7, forming two distinct lineages: BLV-14 clusters closely with BLV-20 and several Russian strains, while BLV-04 appears on a separate branch within the same genotype. In addition, other Kazakhstani isolates group within genotypes G-4 and G-12 [30]. These results confirm the co-circulation of multiple BLV genotypes in Kazakhstan and provide evidence for regional genetic diversity of the virus. Bootstrap values support the robustness of major nodes, including strong clustering of Kazakhstani G-4 and G-7 isolates.

A total of 42 nucleotide sequences were included in this phylogenetic analysis. The codon positions included were the 1st, 2nd, and 3rd positions, as well as non-coding regions. All positions containing gaps or missing data were eliminated using the complete deletion option, resulting in a final dataset of 299 aligned positions. Evolutionary analyses were performed using the maximum likelihood (ML) method under the Tamura–Nei model implemented in MEGA11.

BLASTn (NCBI BLAST v2.14.1, accessed on 15 June 2025) analysis of the three Kazakhstani BLV isolates revealed high nucleotide identity with strains circulating in neighboring regions. Sample No. 4 (BLV-04) demonstrated 99.7% identity with BLV strain BLV-XJ-87 (GenBank: MN765155.1) from the Xinjiang Uygur Autonomous Region, China. Sample No. 14 (BLV-20) showed 99.74% identity with a strain from Tatarstan, Russia (GenBank: KC867319.1). Sample No. 20 (BLV-14) exhibited 100% identity with BLV isolate Tatarsk 23 (GenBank: OP850721.1) from Novosibirsk Region, Russia.

Phylogenetic analysis showed that all three Kazakhstani isolates—BLV-04, BLV-14, and BLV-20—belong to genotype G-7, clustering with strains from Russia, Ukraine, China, and Italy. Within genotype G-7, BLV-14 and BLV-20 formed well-supported cluster, while BLV-04 was placed on a distinct branch, yet still within the G-7 clade.

These findings highlight the genetic proximity between Kazakhstani isolates and those from bordering countries, suggesting cross-border transmission and livestock movement. The presence of genotype G-7 in East Kazakhstan, closely related to strains from Russia and China, underscores the epidemiological significance of regional livestock trade routes and the importance of enhanced transboundary surveillance and collaborative control strategies.

## 4. Discussion

This nationwide surveillance study provides a comprehensive and updated understanding of the epidemiological landscape of BLV in Kazakhstan, integrating serological, molecular, and genetic data collected over a decade. Our findings confirm the persistent endemicity of BLV in several northern and eastern regions and highlight the importance of combining traditional serological techniques with molecular tools for the early detection and characterization of the virus.

The AGID method, recommended by the WOAH as a standard tool for BLV diagnosis, formed the basis for longitudinal seroepidemiological assessment in this study. Over 433,000 serum samples were analyzed, with a national average seroprevalence of 5.87%, and regional peaks exceeding 13% in Kostanay and 16% in North Kazakhstan. These regional disparities likely reflect differences in animal movement, biosecurity practices, and herd management systems. The high prevalence in North Kazakhstan and Kostanay is of particular concern given their proximity to international borders and their role as key livestock-producing zones.

In 2024, a more focused investigation using both AGID and ELISA revealed that ELISA consistently reported higher seroprevalence rates (10.4%) compared to AGID (8.2%), with statistically significant differences (Wilcoxon test, *p* = 0.026). The strong correlation between the methods (Pearson’s r = 0.97) confirms their general agreement; however, the superior sensitivity of ELISA underscores its suitability for detecting low-titer or subclinical infections. These findings are in line with earlier research reporting enhanced diagnostic sensitivity of ELISA for early-stage BLV detection [31,32]. However, it is important to note that higher seroprevalence values do not definitively confirm superior sensitivity, as they may also reflect lower specificity. In this study, a limited subset of ELISA-positive but AGID-negative samples was tested by real-time PCR, and BLV proviral DNA was detected in several of these cases. This molecular confirmation suggests true infection and highlights the potential added value of ELISA in identifying animals with low antibody titers or early-stage infection. Similar findings have been reported in prior comparative studies evaluating BLV diagnostics. Nonetheless, further systematic testing of discrepant samples with direct methods would be required to confirm these observations on a broader scale. Given the lack of a vaccine, early and accurate identification of infected animals remains the cornerstone of BLV control.

A major outcome of this study is the validation of a locally produced AGID test system developed by KazSRVI. The test showed 100% concordance with two international commercial kits (IDEXX and IDvet), even when evaluated across diverse age groups and cattle breeds. This demonstrates that the KazSRVI AGID kit is a reliable and cost-effective alternative for large-scale national surveillance. Its local production may facilitate broader implementation across Kazakhstan and neighboring countries, particularly in rural or resource-limited areas where access to commercial reagents is restricted.

Molecular testing by real-time PCR detected active BLV infection in 4.7% of whole blood samples collected from 12 regions in 2024. Notably, East Kazakhstan and Abai regions accounted for 80% of positive cases, reflecting active viral circulation. These results indicate that despite moderate seroprevalence levels in these areas, BLV-infected animals—regardless of clinical stage—remain lifelong carriers and reservoirs of the virus. In fact, this can contribute to horizontal and vertical transmission of BLV. The detection of BLV proviral DNA in regions with both high and moderate seroprevalence further supports the inclusion of molecular diagnostics in surveillance strategies, especially for identifying epidemiological hotspots and managing latent infections.

Nested PCR and subsequent sequencing of *env* gene fragments from three selected samples revealed that all isolates belonged to genotype G-7. The Kazakhstani isolates exhibited ≥99.7% sequence identity with strains previously reported in China and Russia, suggesting cross-border transmission and shared epidemiological origins. This is consistent with prior studies indicating that genotype G-7 circulates in Eastern Europe and parts of Central Asia [33,34]. The phylogenetic clustering of Kazakhstani isolates with those from neighboring countries reinforces the need for international cooperation in BLV monitoring and control, especially in transboundary livestock trade.

The lack of an effective vaccine against BLV presents a major challenge in limiting the spread of the virus. Thus, control strategies must rely on accurate diagnosis, culling of high-risk or persistently infected animals, and the implementation of biosecurity measures. Our findings support the adoption of a combined diagnostic approach using both ELISA and PCR to identify and manage all BLV-infected animals, including both seropositive and provirus-positive individuals. As BLV persists lifelong in infected cattle, even in the absence of active viral replication, such animals constitute a continual risk for virus transmission. Therefore, the identification of a regional source of infection—regardless of clinical stage—should inform risk-based surveillance and targeted control strategies.

This study represents one of the most extensive assessments of BLV in Central Asia to date. However, it has certain limitations. The reliance on voluntarily submitted samples may introduce bias related to herd type and size. Also, the genetic analysis was limited to a small number of isolates. Further work is needed to expand the molecular surveillance and to determine the relationship between viral genotype, proviral load, and clinical progression.

## 5. Conclusions

This study provides comprehensive insight into the serological epidemiology of bovine leukemia virus (BLV) in Kazakhstan over a 10-year period, complemented by molecular data generated in 2024. Our findings confirm the continued endemicity of BLV in several regions, with notable hotspots of active viral circulation in the east of the country. ELISA identified a higher number of seropositive animals than AGID in this dataset. While this may suggest greater sensitivity, it may also be due to reduced specificity. Limited PCR detection of BLV proviral DNA in ELISA-positive/AGID-negative samples confirmed the presence of latently infected cells. Although such cases may not reflect active viral replication at the time of sampling, these animals remain persistently infected throughout life and can contribute to viral transmission. This underscores the need for cautious interpretation and further validation using complementary diagnostic approaches.

Importantly, the successful validation of a domestically developed AGID test system offers a reliable and cost-effective alternative to imported kits, supporting national surveillance and control efforts. Phylogenetic analysis confirmed the presence of genotype G-7 in Kazakhstan; the identified strains were closely related to isolates from China and Russia, highlighting the importance of cross-border surveillance and cooperation.

In the absence of effective vaccines, integrated diagnostic approaches combining serological and molecular methods remain essential for early detection and containment. These results serve as a foundation for evidence-based policy, risk-based monitoring, and regional collaboration to mitigate the spread of BLV.

## Figures and Tables

**Table 1:** BLV seroprevalence in cattle across different regions of Kazakhstan (2014–2024).

No	Region	Animal Type	Investigated (2014–2024)	Detected	Seroprevalence (%)	95% CI
1	Akmola	Cattle	40,103	1396	3.48	3.31–3.66
2	Aktobe	Cattle	28,596	72	0.25	0.20–0.32
3	Almaty	Cattle	82,704	607	0.73	0.68–0.79
4	Atyrau	Cattle	3678	151	4.11	3.51–4.80
5	Zhambyl	Cattle	40,103	1317	3.28	3.11–3.46
6	East Kazakhstan	Cattle	46,589	3909	8.39	8.14–8.65
7	West Kazakhstan	Cattle	18,436	1037	5.62	5.30–5.97
8	Kyzylorda	Cattle	3687	0	0.00	0.00–0.00
9	Karaganda	Cattle	12,349	56	0.45	0.35–0.59
10	Kostanay	Cattle	52,960	7183	13.56	13.27–13.86
11	Mangystau	Cattle	39	0	0.00	0.00–0.00
12	Pavlodar	Cattle	33,070	2211	6.69	6.42–6.96
13	North Kazakhstan	Cattle	43,007	7220	16.79	16.44–17.14
14	Turkestan	Cattle	17,011	117	0.69	0.57–0.82
15	Ulytau	-	n/a *	-	-	-
16	Abai	Cattle	7790	174	2.23	1.93–2.59
17	Zhetysu	Cattle	3415	0	0.00	0.00–0.00
Total (Republic of Kazakhstan)			433,537	25,450	5.87	

* Note: n/a—not available data.

**Table 2:** Regional distribution of bovine leukemia virus seropositivity in Kazakhstan in 2024 based on AGID screening.

No.	Region	Tested Samples	Positive Results (*n*)	Seroprevalence (%)	95% CI
1	Akmola	171	0	0.0	(0.0–2.2)
2	Almaty	309	32	10.3	(7.43–14.25)
3	Zhetysu	465	0	0.0	0.0–0.82)
4	Atyrau	47	3	6.3	(2.19–17.16)
5	East Kazakhstan	135	12	8.8	(5.16–14.89)
6	Abai	27	6	22.2	(10.61–40.76)
7	Zhambyl	235	3	1.2	(0.44–3.69)
8	Kostanay	276	19	6.8	(4.45–10.5)
9	Aktobe	89	1	1.1	(3.13–13.94)
10	West Kazakhstan	260	9	3.4	(1.83–6.45)
11	Karaganda	235	2	0.8	(0.23–3.05)
12	Ulytau	221	3	1.3	(3.47–9.8)
13	Kyzylorda	251	28	11.1	(7.83–15.65)
14	Mangystau	88	2	2.2	(0.63–7.91)
15	Pavlodar	225	0	0.0	(0.24–3.18)
16	North Kazakhstan	430	176	40.9	(36.38–45.64)
17	Turkestan	272	11	4.0	(2.27–7.1)
Total (Republic of Kazakhstan)		3736	307	8.2	

**Table 3:** Seroprevalence of bovine leukemia virus antibodies in the cattle population categorized by age and breed group.

Regions of the Republic of Kazakhstan	Age (Years)/Breed of Cattle	Number of Tested Samples (*n* Tested)	Diagnostic Systems		
			ID VET (*n* Positive)	IDEXX (*n* Positive)	Diagnostic AGID kit of “KazSRVI” LLP (*n* Positive)
East Kazakhstan region	3–9/Simmental cattle	100	20	20	20
	3–4/Simmental cattle	100	36	36	36
	3–10/Simmental cattle	100	26	26	26
Abai region	3–11/Simmental cattle	100	16	16	16
Abai region	1–8/Kazakh Whiteheaded cattle	60	0	0	0
Kostanay region	5–6/outbred cattle	40	0	0	0
	4–8/Kazakh Whiteheaded cattle	28	12	12	12
	5–7/Kazakh Whiteheaded cattle	60	37	37	37
	4–5/Black-and-white cow breeds	26	9	9	9
	3–7/outbred cattle	87	49	49	49

**Table 4:** Results of real time PCR, AGID, and ELISA testing for bovine leukemia virus in 536 blood samples from Kazakhstan (2024).

No.	Name of Region	Samples Tested	Positive by PCR	AGID Positive	ELISA Positive
1	Akmola	17	0	0	0
2	Almaty	110	3	10	10
3	Zhetysu	130	0	0	0
4	Atyrau	n/a *	n/a	n/a	n/a
5	East Kazakhstan	27	15	10	11
6	Abai	26	5	6	8
7	Zhambyl	60	0	6	6
8	Kostanay	n/a	n/a	n/a	n/a
9	Aktobe	23	0	1	1
10	West Kazakhstan	n/a	n/a	n/a	n/a
11	Karaganda	24	2	2	2
12	Ulytau	22	0	3	4
13	Kyzylorda	26	0	26	26
14	Mangystau	n/a	n/a	n/a	n/a
15	Pavlodar	45	0	0	0
16	North Kazakhstan	n/a	n/a	n/a	n/a
17	Turkestan	26	0	25	25
	Total	536	25	89	93

* n/a—not available; testing was not performed on samples from this region.

## Data Availability

All nucleotide sequences obtained in this study are publicly available in the GenBank database under accession numbers PV648325 (BLV_04_Kazakhstan), PV648326 (BLV_14_Kazakhstan), and PV648327 (BLV_20_Kazakhstan).

## Abbreviations

The following abbreviations are used in this manuscript:

EBL	Enzootic Bovine Leukosis
AGID	Agar Gel Immunodiffusion
BLAST	Basic Local Alignment Search Tool
BLV	Bovine Leukemia Virus
bp	Base Pairs
Ct	Cycle Threshold
DNA	Deoxyribonucleic Acid
ELISA	Enzyme-Linked Immunosorbent Assay
env	Envelope Gene
ML	Maximum Likelihood
NTC	No Template Control
OD	Optical Density
PCR	Polymerase Chain Reaction
RT-PCR	Real-Time Polymerase Chain Reaction
RNA	Ribonucleic Acid
SPSS	Statistical Package for the Social Sciences
WOAH	World Organisation for Animal Health (formerly OIE)

## References

[1] Bartlett P.C., Sordillo L.M., Byrem T.M., Norby B., Grooms D.L., Swenson C.L., Zalucha J., Erskine R.J. (2014). Options for the Control of Bovine Leukemia Virus in Dairy Cattle. Javma.

[2] Marawan M.A., Alouffi A., El Tokhy S., Badawy S., Shirani I., Dawood A., Guo A., Almutairi M.M., Alshammari F.A., Selim A. (2021). Bovine Leukaemia Virus: Current Epidemiological Circumstance and Future Prospective. Viruses.

[3] Mamanova S., Kaimoldina S., Kassen A., Nissanova R., Bashenova E. (2025). Study of Circulation of Bovine Leukaemia Virus Genotypes in Central Asia. CABI Rev..

[4] Asfaw Y., Sentsui H., Murakami K., Tsuduku S., Tsuboi T., Konishi M., Wu D. (2004). Distribution and Superinfection of Bovine Leukemia Virus Genotypes in Japan. Arch. Virol..

[5] Nobrega D.B., Miltenburg C., Séguin G., Kelton D.F. (2024). Prevalence and Spatial Distribution of Infectious Diseases of Dairy Cattle in Ontario, Canada. J. Dairy Sci..

[6] Hamada R., Metwally S., Polat M., Borjigin L., Ali A.O., Abdel-Hady A.A.A., Mohamed A.E.A., Wada S., Aida Y. (2020). Detection and Molecular Characterization of Bovine Leukemia Virus in Egyptian Dairy Cattle. Front. Vet. Sci..

[7] Shrestha S., Orsel K., Barkema H.W., Martins L., Shrestha S., Meer F.V.D. (2023). Effects of Bovine Leukemia Virus Seropositivity and Proviral Load on Milk, Fat, and Protein Production of Dairy Cows. J. Dairy Sci..

[8] Bai L., Borjigin L., Sato H., Takeshima S.-N., Asaji S., Ishizaki H., Kawashima K., Obuchi Y., Sunaga S., Ando A. (2021). Kinetic Study of BLV Infectivity in BLV Susceptible and Resistant Cattle in Japan from 2017 to 2019. Pathogens.

[9] Frie M.C., Coussens P.M. (2015). Bovine Leukemia Virus: A Major Silent Threat to Proper Immune Responses in Cattle. Vet. Immunol. Immunopathol..

[10] Borjigin L., Watanuki S., Hamada R., Bai L., Hirose T., Sato H., Yoneyama S., Yasui A., Yasuda S., Yamanaka R. (2023). Effectiveness of Integrated Bovine Leukemia Virus Eradication Strategies Utilizing Cattle Carrying Resistant and Susceptible Major Histocompatibility Complex Class II DRB3 Alleles. J. Dairy Sci..

[11] Nakatsuchi A., Sato R., Borjigin L., Takeshima S.-N., Sato H., Watanuki S., Aida Y., Bai L., Murakami H., Asaji S. (2022). BoLA-DRB3 Polymorphism Controls Proviral Load and Infectivity of Bovine Leukemia Virus (BLV) in Milk. Pathogens.

[12] Borjigin L., Takeshima S.-N., Yasuda S., Tanaka N., Yamanaka R., Shinozaki Y., Lo C.-W., Sato H., Yasui A., Bai L. (2021). Risk Assessment of Bovine Major Histocompatibility Complex Class II DRB3 Alleles for Perinatal Transmission of Bovine Leukemia Virus. Pathogens.

[13] Kobayashi S., Konishi M., Yamamoto T., Kameyama K.-I., Hayama Y., Tsutsui T., Murakami K. (2010). Risk Factors Associated with Within-Herd Transmission of Bovine Leukemia Virus on Dairy Farms in Japan. BMC Vet. Res..

[14] Pereira J.G., de Assunção Silva C., Silva L.D., Lima C.A.A., do Rosário C.J.R.M., Silva E.M.C., do Socorro Costa Oliveira Oliveira M., dos Santos Ribeiro L.S., Santos H.P., Abreu-Silva A.L. (2023). Diagnosis and Phylogenetic Analysis of Bovine Leukemia Virus in Dairy Cattle in Northeastern Brazil. Front. Vet. Sci..

[15] Úsuga-Monroy C., Díaz F.J., González-Herrera L.G., Echeverry-Zuluaga J.J., López-Herrera A. (2023). Phylogenetic Analysis of the Partial Sequences of the Env and Tax BLV Genes Reveals the Presence of Genotypes 1 and 3 in Dairy Herds of Antioquia, Colombia. VirusDisease.

[16] Mendoza W., Isaza J.P., López L., López-Herrera A., Gutiérrez L.A. (2023). *Bovine leukemia virus* Detection in Humans: A Systematic Review and Meta-Analysis. Virus Res..

[17] Bartlett P.C., Taxis T.M., Droscha C.J., Hutchinson H.C., Norby B., Sporer K.R.B., Ruggiero V.J. (2020). Current Developments in the Epidemiology and Control of Enzootic Bovine Leukosis as Caused by Bovine Leukemia Virus. Pathogens.

[18] Jaworski J.P., Willems L., Vahlenkamp T.W., Alvarez I., Rola-Łuszczak M., Carignano H.A., Murakami K., Kuźmak J., Pluta A., Choudhury B. (2018). Interlaboratory Comparison of Six Real-Time PCR Assays for Detection of Bovine Leukemia Virus Proviral DNA. J. Clin. Microbiol..

[19] FAOSTAT. https://www.fao.org/faostat/en/#data/PP.

[20] Sultanov A., Rola-Łuszczak M., Mamanova S., Ryło A., Osiński Z., Saduakassova M.A., Bashenova E., Kuźmak J. (2022). Molecular Characterization of Bovine Leukemia Virus with the Evidence of a New Genotype Circulating in Cattle from Kazakhstan. Pathogens.

[21] Thrusfield M.V. (2008). Veterinary Epidemiology.

[22] WOAH—World Organisation for Animal Health Codes and Manuals. https://www.woah.org/en/what-we-do/standards/codes-and-manuals/.

[23] Fechner H., Kurg A., Geue L., Blankenstein P., Mewes G., Ebner D., Beier D. (1996). Evaluation of Polymerase Chain Reaction (PCR) Application in Diagnosis of Bovine Leukaemia Virus (BLV) Infection in Naturally Infected Cattle. J. Vet. Med. Ser. B.

[24] Rola-Łuszczak M., Finnegan C., Olech M., Choudhury B., Kuźmak J. (2013). Development of an Improved Real Time PCR for the Detection of Bovine Leukaemia Provirus Nucleic Acid and Its Use in the Clarification of Inconclusive Serological Test Results. J. Virol. Methods.

[25] Tamura K., Stecher G., Kumar S. (2021). MEGA11: Molecular Evolutionary Genetics Analysis Version 11. Mol. Biol. Evol..

[26] KazNIVI Bovine Leukemia Virus Isolate BLV-20 Envelope Glycoprotein (Env) Gene, Partial Cds 2025. GenBank: PV648327.1. https://www.ncbi.nlm.nih.gov/nuccore/PV648327.1.

[27] KazNIVI Bovine Leukemia Virus Isolate BLV-14 Envelope Glycoprotein (Env) Gene, Partial Cds 2025. GenBank: PV648326.1. https://www.ncbi.nlm.nih.gov/nuccore/PV648326.1.

[28] KazNIVI Bovine Leukemia Virus Isolate BLV-04 Envelope Glycoprotein (Env) Gene, Partial Cds 2025. GenBank: PV648325.1. https://www.ncbi.nlm.nih.gov/nuccore/PV648325.1.

[29] Thiermann A.B., Babcock S. (2021). Animal Welfare and International Trade.

[30] Trono K.G., Pérez-Filgueira D.M., Duffy S., Borca M.V., Carrillo C. (2001). Seroprevalence of Bovine Leukemia Virus in Dairy Cattle in Argentina: Comparison of Sensitivity and Specificity of Different Detection Methods. Vet. Microbiol..

[31] Monti G.E., Frankena K., Engel B., Buist W., Tarabla H.D., de Jong M.C.M. (2005). Evaluation of a New Antibody-Based Enzyme-Linked Immunosorbent Assay for the Detection of Bovine Leukemia Virus Infection in Dairy Cattle. J. Vet. Diagn. Investig..

[32] Pluta A., Rola-Łuszczak M., Kubiś P., Balov S., Moskalik R., Choudhury B., Kuźmak J. (2017). Molecular Characterization of Bovine Leukemia Virus from Moldovan Dairy Cattle. Arch. Virol..

[33] Ochirkhuu N., Konnai S., Odbileg R., Nishimori A., Okagawa T., Murata S., Ohashi K. (2016). Detection of Bovine Leukemia Virus and Identification of Its Genotype in Mongolian Cattle. Arch. Virol..

[34] Wang L., Ning C., Ji C., Guo Y., Li N., Qiao J., Meng Q., Xia X., Zhang X., Liu Y. (2021). Detection and Genetic Characteristics of Bovine Leukaemia Virus in Holstein Cows in China. Int. J. Agric. Biol..

